# Environmental drivers of Cheirogaleidae population density: Remarkable resilience of Madagascar’s smallest lemurs to habitat degradation

**DOI:** 10.1002/ece3.7449

**Published:** 2021-05-02

**Authors:** Daniel Hending

**Affiliations:** ^1^ School of Biological Sciences The University of Bristol Bristol UK; ^2^ Bristol Zoological Society Clifton, Bristol UK

**Keywords:** adaptability, Cheirogaleidae, habitat degradation, lemur, Madagascar, meta‐analysis, protected areas

## Abstract

**Aim:**

Global animal populations are in decline due to destruction and degradation of their natural habitat. Understanding the factors that determine the distribution and density of threatened animal populations is therefore now a crucial component of their study and conservation. The Cheirogaleidae are a diverse family of small‐bodied, nocturnal lemurs that are widespread throughout the forests of Madagascar. However, many cheirogaleid lemurs are now highly threatened with extinction and the environmental factors that determine their distribution and population density are still little known. Here, I investigated the environmental drivers of Cheirogaleidae population density at genus level.

**Location:**

Various forest sites across Madagascar.

**Methods:**

I investigated how six environmental variables affect Cheirogaleidae population density at the genus level via random‐effect meta‐analyses. I then used a generalized linear mixed‐effects model to identify the primary predictors of Cheirogaleidae population density. Finally, I investigated how the population density of this family of lemurs varies between protected and unprotected areas of Madagascar via a GLM analysis.

**Results:**

My results indicate that the relationships between the tested environmental factors and population density are genus‐specific among the Cheirogaleidae. Rather remarkably, the density of *Microcebus* appears to have a profoundly positive relationship with anthropogenic disturbance and a negative relationship with forest cover, a finding that is also reflected by larger population densities within unprotected areas in comparison with localities within Madagascar's protected area network.

**Main Conclusions:**

The results of this study are highly encouraging for the conservation of the Cheirogaleidae and highlight the remarkable resilience of these lemurs to habitat degradation and anthropogenic activity. However, this study also outlines the dearth of knowledge that we have for many species, and why these data are urgently needed to understand the biogeography and ecology of threatened animal populations and implement successful conservation.

## Graphical abstract

In this manuscript, I investigated the primary environmental drivers of population density in the Cheirogaleidae, a family of small, nocturnal, and understudied primates that are endemic to Madagascar. My results indicate that the relationships between the tested environmental factors and population density are genus‐specific among the Cheirogaleidae, and this is likely due to the natural histories and ecological niches of each respective genus. Further, forest quality and anthropogenic disturbance do not appear to negatively influence Cheirogaleidae density, which remains high outside of protected areas; this is a highly encouraging finding for this group of threatened primates.

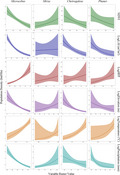

## 1 INTRODUCTION

Global animal populations face geographic range contraction and localized extinction (Cardillo et al., 2005; Channell & Lomolino, 2000) due to the ongoing destruction and anthropogenic disturbance of natural habitats (Fahrig, 1997; Goldammer, 2013). Understanding the distribution, density and composition of global animal populations, and the factors that determine them is therefore now a crucial component of the study of animal ecology, evolution, and natural history (Andrewartha & Birch, 1954; Dempster, 1975). Further, a detailed knowledge of the determinants and limiters of animal distributions and densities is required at both the population and species‐specific level for the effective management and conservation of the remaining populations of threatened taxa (Karanth et al., 2009; Scott, 1988; Sibly & Hone, 2002). Population densities are often governed by a number of biotic mechanisms, including interspecific and intraspecific resource competition (Gurevitch et al., 2000; Tilman, 1982; Wise, 2006), predation (Thirgood et al., 2000), vegetation availability (Layme et al., 2004), habitat quality (Caughley, 1977), and pathogens and disease (Cully et al., 2010). Some of these biotic factors, such as habitat quality and availability, often have a positive effect on population densities (Johnson & Arcata, 2005), whereas density is often negatively affected and often limited by other factors such as disease and competition (Tilman, 1982). Abiotic factors such as climate (Fischer et al., 2001), elevation (Lomolino, 2001), and geography (Gaston, 2009) also play a fundamental role in determining species density, and the correlation of density with such variables are determined by the species’ ecological niche preference (Hutchingson, 1957). The disparities in population density between different taxa and subpopulations are often due to interactions of both biotic and abiotic mechanisms (e.g., Buckley & Jetz, 2007; Lewis et al., 2017), and a sound understanding of the relative influence of each of these elements is therefore essential to clarify the determinants of species demography, and gage the potential extinction risk for threatened taxa (Davidson et al., 2009; Schurr et al., 2012; Sinclair & Byrom, 2006).

The lemurs of Madagascar are a diverse group of over 100 primate species that are regarded as one of the most threatened groups of mammals in the world (Mittermeier et al., 2008; Schwitzer et al., 2013). Due to Madagascar's variable topography and extensive network of waterways, microclimatic and biogeographic zonation persists throughout the island and lemur distribution is often restricted and constrained by these natural barriers (Brown et al., 2016; Tattersall & Sussman, 1975; Wilmé et al., 2006). The distribution of many lemur species is therefore largely confined to areas of localized microendemism (Markolf & Kappeler, 2013; Wilmé et al., 2006) and the diversity and density of lemur populations is highly variable throughout Madagascar (Setash et al., 2017). While the ecological determinants of population density are unstudied for many lemur species (Ganzhorn et al., 2006; Mittermeier et al., 2010), several investigations have proposed a range of theories to explain the discrepancies in population density between the taxa for which data exists. For instance, lemur population density has been observed to vary greatly between Madagascar's various forest types (Axel & Maurer, 2011), with some studies revealing higher populations in the dry forests of western regions than in the humid forests of the east (Ganzhorn et al., 2006; Setash et al., 2017), and higher densities in forest interior habitat than in forest edges (Lehman et al., 2006a). Habitat degradation and disturbance have also been demonstrated to have mostly negative effects on the population density of lemurs (Ganzhorn et al., 1997; Lehman et al., 2006a), and the population responses to these anthropogenic drivers are often reported as species‐specific (Eppley et al., 2020; Herrera et al., 2011; Lehman et al., 2006b; Steffens et al., 2020). High population density variation has been observed along several environmental gradients, and there is evidence of both positive and negative correlations between lemur density and elevation (Campera et al., 2020; Goodman & Ganzhorn, 2004) and negative correlations with water availability (Axel & Maurer, 2011). There is also evidence to suggest that optimal climatic conditions (Kamilar et al., 2016), vegetation quality and productivity (Ganzhorn, 1995), and food availability (Steffens & Lehman, 2016) may also positively influence lemur population density. These previous investigations suggest that lemur population densities are shaped by a range of both biotic and abiotic factors in what is likely a complex process.

The Cheirogaleidae are a diverse family of lemurs, made up of five genera, containing a total of 41 small‐bodied, nocturnal, and largely solitary species (Hotaling et al., 2016; McLain et al., 2017; Mittermeier et al., 2008). Cheirogaleid lemurs are widespread throughout all of Madagascar's forest types and they are present within both pristine and disturbed habitat (Mittermeier et al., 2010). Multiple species of cheirogaleid lemurs often live in sympatry with each other (e.g., Lahann, 2008; Rakotondranary & Ganzhorn, 2011), and there is strong evidence of ecological niche separation between genera, and in some cases between species (Kamilar et al., 2016; Lahann, 2007; Rakotondravony & Radespiel, 2009). In comparison with other lemurs, some Cheirogaleidae are surprisingly resilient and adaptable to environmental change and habitat disturbance (Kappeler & Rasoloarison, 2003; Lehman et al., 2016), and some species have been observed to inhabit highly degraded, anthropogenic habitats such as gallery forests, agroecosystems, and even gardens (Ganzhorn, 1987; Hending et al., 2018; Mittermeier et al., 2010). The large geographic distribution of the cheirogaleids, their presence in various habitat types, and the variations in their biogeography, ecology, and adaptability to heterogeneous ecological conditions (some species are more specialized than others) make them an ideal model in which we can further investigate the biotic and abiotic determinants of population density and how population responses to environmental conditions vary between closely related animals (Steffens & Lehman, 2016). Further, many of the Cheirogaleidae are now highly threatened with extinction and a detailed knowledge of their population dynamics is critical for an informed understanding of their biogeography and for the implementation of effective conservation (Schwitzer et al., 2013; Steffens & Lehman, 2018).

In this study, I aimed to determine the primary drivers of population density of each of the five Cheirogaleidae genera using density data published within the literature. I opted to use a genus‐level approach in this investigation as the general ecology and natural history of the species within genera are highly similar (Lehman et al., 2016; Mittermeier et al., 2010; Radespiel, 2006), and each lemur genus has a distinct ecological niche (e.g., Campera et al., 2019; Kamilar & Muldoon, 2010; Lahann, 2008); while niche separation and divergence does exist among sympatric congeners on an often local or site‐specific scale (e.g., Dammhahn & Kappeler, 2008; Lahann, 2007, 2008; Radespiel et al., 2003; Rakotondravony & Radespiel, 2009; Thorén et al., 2011), the broad effect of biogeography and environmental variables on lemurs over a large area often follows a trend (e.g., Campera et al., 2020; Herrera, 2017; Pearson & Raxworthy, 2009; Setash et al., 2017). The specific objectives of this study were:

1. First, to explore the relationship between population density of the five Cheirogaleidae genera and six environmental variables. As different lemur groups have often been observed to respond interspecifically to different environmental variables (e.g., Campera et al., 2020; Herrera et al., 2011; Kamilar et al., 2016; Lehman et al., 2006b), I hypothesized that relationships between population density and the environmental variables would be highly specific to each of the five Cheirogaleidae genera. As all lemurs require forest habitat for their survival and many species are sensitive to habitat degradation (Schwitzer et al., 2013), I predicted that population density of all genera would correlate positively with vegetation‐related variables and negatively with anthropogenic disturbance. However, due to the results of previous studies (Campera et al., 2020; Kamilar et al., 2016; Setash et al., 2017), I also predicted that population density would correlate positively with the abiotic variables temperature and precipitation, and negatively with elevation.
2. Second, to identify the primary environmental drivers of population density for each of the cheirogaleid genera. I hypothesized that vegetation‐related variables would be the primary drivers of population density for all genera, as per my previous hypothesis for objective one. As many Cheirogaleid species have been observed living within highly degraded and anthropogenic habitat types (Hending et al., 2018; Kappeler & Rasoloarison, 2003; Webber et al., 2020), I also predicted that anthropogenic disturbance would not be a primary density driver.
3. Third, to compare how population densities vary between Madagascar's protected area system and unprotected areas for each cheirogaleid genus, in order to gain insight into the importance of the protected area network for their conservation. Population declines of all lemurs have been observed in recent years, and these declines are hypothesized to be attributed primarily to forest loss (Schwitzer et al., 2013; Schwitzer et al., 2014; IUCN, 2020), so I predicted that the population densities of all genera would be significantly higher within protected areas than in unprotected areas.

## 2 METHODS

### 2.1 Literature Review

I compiled a database of Cheirogaleidae population density values that I obtained during a search of the literature. To find the relevant published material, I searched the full volume catalogue of several journals that frequently publish studies on lemur populations, including the International Journal of Primatology, American Journal of Primatology, Folia Primatologica, Primates, Primate Conservation, Lemur News, Madagascar Conservation and Development, and Malagasy Nature. I opted for this rather time‐consuming method because literature databases often do not contain studies published in local or regional journals (Lemur News, Malagasy Nature etc.) and although this search method was very laborious, it ensured that I did not miss any important studies. In addition to these specific journals, I also searched the literature databases Google Scholar, JSTOR, Science Direct, Springer Link, Wiley, Web of Science, and ResearchGate for articles published in other scientific journals, edited book volumes, and dissertations. I used the keywords “population,” “density,” “survey,” “lemur” and “Cheirogaleidae” in my literature search, in addition to the taxa‐specific keywords “mouse lemur,” “dwarf lemur,” “giant mouse lemur,” “fork‐marked lemur,” “*Microcebus*,” “*Cheirogaleus*,” “*Mirza*,” “*Allocebus*” and “*Phaner*.” I included both primary literature, such as journal articles and book chapters, and gray literature, such as unpublished theses and unpublished reports, as part of my review as both literature types contain important information pertaining to lemur population size and density. I initially included studies that report population encounter rates (e.g., *N*/km) as well as those that report true population density values (e.g., *N*/Ha) in my database. However, I did not include papers that use proxy population density values (e.g., biomass estimates: Simmen et al., 2012, acoustic survey estimates: Hending, Holderied et al., 2017; Hending et al., 2020) as these are not comparable with true density values or encounter rates. A list of all data sources is found in Appendix [Link], [Link], [Link], [Link], [Link].

Many publications contained density data for multiple species and for several different sites. Also, several population density values often existed in the literature for a single species, either from the same locality or from a different location. I included all values from all studies as separate data points in my database for analysis. For all population density records in my database, I recorded the specific GPS coordinates of the study to as many decimal places as possible (depending on what was provided in each publication), the corresponding species and genus, and their conservation status. I updated the species names in my database to reflect the current Cheirogaleidae taxonomy using the geographic location of the respective study and the species distribution information available in the most recent lemur Red List assessments (IUCN, 2020), as many of the studies in my database were conducted prior to recent species descriptions. I also noted the forest type in which the study took place (humid, dry, transitional, or spiny: Chauvet, 1972), the season in which the study was conducted (wet, dry or both), the sampling method that was used (transect distance sampling or trapping/capture–recapture), and the method used to calculate the density value. Finally, I noted whether the study locality was within a protected area (National Park, Special Reserve, Protected Area, Classified Forest etc.) using the precise GPS locality of the study, the most up to date literature on the protected areas of Madagascar (Goodman et al., 2018), and a freely available raster layer of Madagascar's protected area network (UNEP‐WCMC, 2020).

In total, I found 75 studies in my literature review that report on the population densities and encounter rates of cheirogaleid lemurs. These studies made up a total of 421 data points of which 278 included population density values and 207 included encounter rates (89 data points included both density and encounter rate values). After I had removed the encounter rate‐only data points from the dataset, the 278 data points involved in the analyses were made up of data from a total of 59 studies (Appendix S1). The literature contained the most population density data points for *Microcebus murinus* (*N* = 45), *Cheirogaleus medius* (*N* = 29), and *C. major* (*N* = 29), respectively, while I found no population density data in the literature for 16 species (*M. arnholdi, M. bongolavensis, M. boraha, M. gerpi, M. jollyae, M. macarthurii, M. mamiratra, M. manitatra, M. marohita, C. andysabini, C. grovesi, C. lavasoensis, C. shethi, C. thomasi, Phaner electromontis,* and *P. parienti*). Population density values ranged from 0.01 to 12.72 individuals/Ha across all species (Table 1). I could not identify 14 of the data points to species level (mouse lemurs from the Makira National Park and Masoala peninsula). *M. mittermeieri* and *M. macarthurii* both live sympatrically within Makira, and it is not clear as to which species the density values in the literature refer to (Schüßler et al., 2020); I have therefore included the Makira mouse lemurs in Table 1 as “*Microcebus* spp.” The species identity of the Masoala mouse lemurs is also not known, so I have henceforth grouped them as “*Microcebus* sp. 2” in Table 1.

**TABLE 1:** A summary of the population density (*N*/Ha) data available for all species of the Cheirogaleidae family in the literature

Scientific name	Common name	IUCN status	Mean population density (*N*/Ha)	Population density range (*N* /Ha)	Sample size (*N*)	Localities (*N*)	Studies (*N*)
*Microcebus* spp.	*N*/A	*N*/A	0.34	0.04–1.06	12	12	2
*Microcebus* sp. 2	*N*/A	*N*/A	1.35	0.37–2.32	2	2	2
*Microcebus arnholdi*	Arnhold's Mouse Lemur	VU	*N*/A	*N*/A	0	0	0
*Microcebus berthae*	Madame Berthe's Mouse Lemur	CR	2.49	0.34–12.72	13	7	3
*Microcebus bongolavensis*	Bongolava Mouse Lemur	EN	*N*/A	*N*/A	0	0	0
*Microcebus boraha*	Boraha Mouse Lemur	DD	*N*/A	*N*/A	0	0	0
*Microcebus danfossi*	Danfoss's Mouse Lemur	VU	3.90	2.41–5.27	14	14	2
*Microcebus ganzhorni*	Ganzhorn's Mouse Lemur	EN	6.80	6.80–6.80	1	1	1
*Microcebus gerpi*	GERP's Mouse Lemur	CR	*N*/A	*N*/A	0	0	0
*Microcebus griseorufus*	Grey‐brown Mouse Lemur	LC	5.27	2.20–11.80	14	6	6
*Microcebus jollyae*	Jolly's Mouse Lemur	EN	*N*/A	*N*/A	0	0	0
*Microcebus jonahi*	Jonah's Mouse Lemur	UN	0.96	0.96–0.96	1	1	1
*Microcebus lehilahytsara*	Goodman's Mouse Lemur	VU	1.63	0.05–3.75	3	3	3
*Microcebus macarthurii*	Macarthur's Mouse Lemur	EN	*N*/A	*N*/A	0	0	0
*Microcebus mamiratra*	Claire's Mouse Lemur	EN	*N*/A	*N*/A	0	0	0
*Microcebus manitatra*	Manitatra Mouse Lemur	CR	*N*/A	*N*/A	0	0	0
*Microcebus margotmarshae*	Margot Marsh's Mouse Lemur	EN	2.20	2.20–2.20	1	1	1
*Microcebus marohita*	Marohita Mouse Lemur	CR	*N*/A	*N*/A	0	0	0
*Microcebus mittermeieri*	Mittermeier's Mouse Lemur	EN	0.07	0.07–0.07	1	1	1
*Microcebus murinus*	Grey Mouse Lemur	LC	3.29	0.29–12.72	45	32	17
*Microcebus myoxinus*	Pygmy Mouse Lemur	VU	3.25	0.57–6.67	5	3	3
*Microcebus ravelobensis*	Golden‐brown Mouse Lemur	VU	3.35	0.08–9.38	18	12	3
*Microcebus rufus*	Brown Mouse Lemur	VU	0.79	0.06–3.90	11	6	7
*Microcebus sambiranensis*	Sambirano Mouse Lemur	EN	1.25	1.25–1.25	1	1	1
*Microcebus simmonsi*	Simmons' Mouse Lemur	EN	1.32	0.62–2.50	3	3	3
*Microcebus tanosi*	Anosy Mouse Lemur	EN	3.00	3.00–3.00	1	1	1
*Microcebus tavaratra*	Northern Rufous Mouse Lemur	VU	1.58	0.29–3.25	12	11	3
*Mirza coquereli*	Coquerel's Giant Mouse Lemur	EN	0.81	0.01–2.10	19	13	7
*Mirza zaza*	Northern Giant Mouse Lemur	VU	3.27	2.68–3.85	2	2	2
*Allocebus trichotis*	Hairy‐eared Dwarf Lemur	EN	0.11	0.07–0.19	5	4	2
*Cheirogaleus andysabini*	Montagne d'Ambre Dwarf Lemur	EN	*N*/A	*N*/A	0	0	0
*Cheirogaleus crossleyi*	Crossley's Dwarf Lemur	VU	0.51	0.02–1.38	6	5	5
*Cheirogaleus grovesi*	Grove's Dwarf Lemur	DD	*N*/A	*N*/A	0	0	0
*Cheirogaleus lavasoensis*	Lavasoa Dwarf Lemur	EN	*N*/A	*N*/A	0	0	0
*Cheirogaleus major*	Greater Dwarf Lemur	VU	0.47	0.08–1.35	29	23	14
*Cheirogaleus medius*	Fat‐Tailed Dwarf Lemur	VU	1.86	0.20–7.50	29	21	10
*Cheirogaleus shethi*	Sheth's Dwarf Lemur	EN	*N*/A	*N*/A	0	0	0
*Cheirogaleus sibreei*	Sibree's Dwarf Lemur	CR	0.07	0.07–0.07	1	1	1
*Cheirogaleus thomasi*	Thomas's Dwarf Lemur	EN	4.2	0.80–12.00	4	4	3
*Phaner electromontis*	Montagne d'Ambre Fork‐marked Lemur	EN	*N*/A	*N*/A	0	0	0
*Phaner furcifer*	Masoala Fork‐marked Lemur	EN	0.07	0.03–0.12	4	3	2
*Phaner pallescens*	Pale Fork‐marked Lemur	EN	1.77	0.16–8.50	21	14	8
*Phaner parienti*	Sambirano Fork‐marked lemur	EN	*N*/A	*N*/A	0	0	0

### 2.2 Extraction of Environmental and Climatic Variables

For each record in my database, I extracted mean values for the Normalized Difference Vegetation Index (NDVI), Leaf Area Index (LAI), and Human Footprint (HFP) using the packages “raster” (Hijmans, 2017), “rgdal” (Bivand et al., 2019), and “sp” (Bivand et al., 2013) in R Studio (R Core Team, 2017). NDVI is a proxy of plant productivity (Rouse et al., 1974), LAI is a common proxy of tree cover density (Asner et al., 2003; Bremond et al., 2005), and HFP is a proxy of anthropogenic disturbance (Venter et al., 2016), all of which have been regularly used to model and analyze mammalian populations in past studies (e.g., Campera et al., 2020; Leyequien et al., 2007; Rodríguez et al., 2006). Density datapoints were obtained from studies conducted 1964–2018 (Appendix [Link], [Link], [Link], [Link], [Link]), and due to recent deforestation in Madagascar (Vieilledent et al., 2018), I checked to ensure that the localities of these data points were still forested using current high‐resolution (<1 m/pixel), cloud‐free, satellite imagery in Google Earth Pro (version 7.3.3, Google LLC, Mountain View CA, USA). I used the geographic coordinates of each data point to extract NDVI and LAI values from monthly TIFF layers (resolution of 1 km^2^) from January 2000 until February 2020 that I downloaded from neo.sci.gsfc.nasa.gov/. I then used these monthly values to calculate a mean NDVI and LAI value for each data point. For HFP, I extracted a mean value for each data point from the latest available TIFF layer (2009, resolution of 1 km^2^) in the data repository of Venter et al. (2016); the distribution of anthropogenic features has changed little over the density datapoint period (1964–2018), as most of the villages and roads that comprise it were established in some form prior to 1964 (Dewar & Wright, 1993; Little, 1884). In addition to NDVI, LAI, and HFP, I extracted elevational data (where not provided in the literature) from the SRTM database (resolution of 90 m^2^, resampled to 1 km^2^) using the “raster” R package. In addition, I also extracted mean annual temperature (Bio1) and annual precipitation (Bio12) climatic data for each data point from the WorldClim database (WorldClim, 2020), which are commonly used in species distribution models (Booth et al., 2014; Nix, 1986). To extract the climatic variables, I used the R package “pscl” (Jackman, 2017) and the most sensitive resolution possible (~1 km^2^). Instead of extracting all 19 bioclimatic variables and using a data‐reduction approach to obtain temperature and precipitation components (such as redundancy analysis or principal component analysis), I opted to instead use the Bio1 and Bio12 values; this is because the directionality of reduced variables would not be clear, making it difficult to determine whether climate had a positive or negative correlation with density.

### 2.3 Analyses

Population encounter rates were much less prominent in the literature in comparison with population density values. I therefore removed the data points for the studies that measured encounter rates only, and I only included actual population density values in my analyses so that values were comparable. To prepare the dataset for meta‐analysis, I used separate Spearman's rank correlation tests to investigate the relationship between population density and NDVI, LAI, HFP, elevation, temperature, and precipitation for each Cheirogaleidae genus. For individual species data points from the same location (GPS point), I calculated a mean density for this locality and included it in the analysis as a single data point. As Spearman's test statistics (*ρ*) are non‐normally distributed, I transformed them into *Z*‐scores (*Z*) and effect sizes (*VZ*) using the formulas:

Z=0.5×ln((1+ρ)) and VZ=1/(N‐3).

I performed a random‐effects meta‐analysis, with a restricted maximum likelihood estimator, on the *Z* and *VZ* values (Borenstein et al., 2011) using the R packages “metafor” (Veichtbauer, 2010) and “robumeta” (Fisher et al., 2017) for each environmental variable (Appendix S2). I chose to use a random‐effects meta‐analysis rather than a fixed‐effects analysis as the random‐effects approach assumes and accounts for differences in study effects (i.e., differences in sampling method and density calculation between data points); confidence intervals are larger, and therefore more conservative in random‐effects meta‐analysis models in comparison with those of fixed‐effects models (Riley et al., 2011). I tested the residual heterogeneity using Q tests, where a significant result indicates that significant variability exists between the effect sizes and that genera do not include common effects. It was not possible to conduct a meta‐analysis on the relationship between population density and forest type, as forest type is a categorical variable and *Z*‐scores and effect sizes could therefore not be computed.

To investigate the primary predictors of population density for the *Microcebus*, *Mirza*, *Cheirogaleus* and *Phaner* genera, I used generalized linear mixed‐effect models (GLMMs) in the R package “lme4” (Bates et al., 2015). I could not perform this type of analysis for the *Allocebus* genus as the sample size of population density values was too small (*N* = 5). Prior to running the GLMMs, I log10 transformed the LAI, HFP, elevation, temperature, and precipitation values so that all continuous variables included in the analysis were of the same order of magnitude. In the GLMM, population density was the response variable, while the independent fixed effects were NDVI, LAI (log10), HFP (log10), elevation (log10), temperature (log10), precipitation (log10), and forest type; forest type was also included in the model to control for variances in lemur detectability between data points collected in the different forest types (Smith et al., 1997). As density estimates vary depending on the sampling methods and density calculation methods used (e.g., Sterling & Ramaroson, 1996), I controlled for differences in sampling method, density calculation method, and season in which the data were collected between the data points by including them in the model as random factors. I then ran the GLMM with a poisson family and selected the best model based on the Akaike information criterion (AIC). As strong correlations and synergistic patterns often exist between environmental variables (Faith & Norris, 1989; Liira et al., 2007), I tested for multicollinearity between the independent variables in each GLMM by calculating Generalized Variance Inflation Factors (GVIFs: Fox & Monette, 1992) with the R package “car” (Fox & Weisberg, 2019); all GVIF values for each independent variable in each GLMM were under 5 (Appendix [Link], [Link], [Link], [Link], [Link]), indicating that no significant amount of multicollinearity existed between the independent variables.

Finally, I compared the mean population densities of *Microcebus*, *Mirza*, *Cheirogaleus,* and *Phaner* between protected areas and unprotected areas using general linear models (GLMs). I used Levene tests in the R package “car” to ensure that the variances of the population densities within each genus were homogenous (Appendix [Link], [Link], [Link], [Link], [Link]). I then ran the GLMs with population density as the dependent variable, an independent grouping variable defining whether the data point was in a protected or unprotected area, and I controlled for season, sampling method, density calculation method, and lemur detectability (forest types) between data points by including them as independent variables. All analyses described in this manuscript were ran with α‐level of 0.05.

## 3 RESULTS

### 3.1 Density Relationship with Individual Variables

**FIGURE 1:**
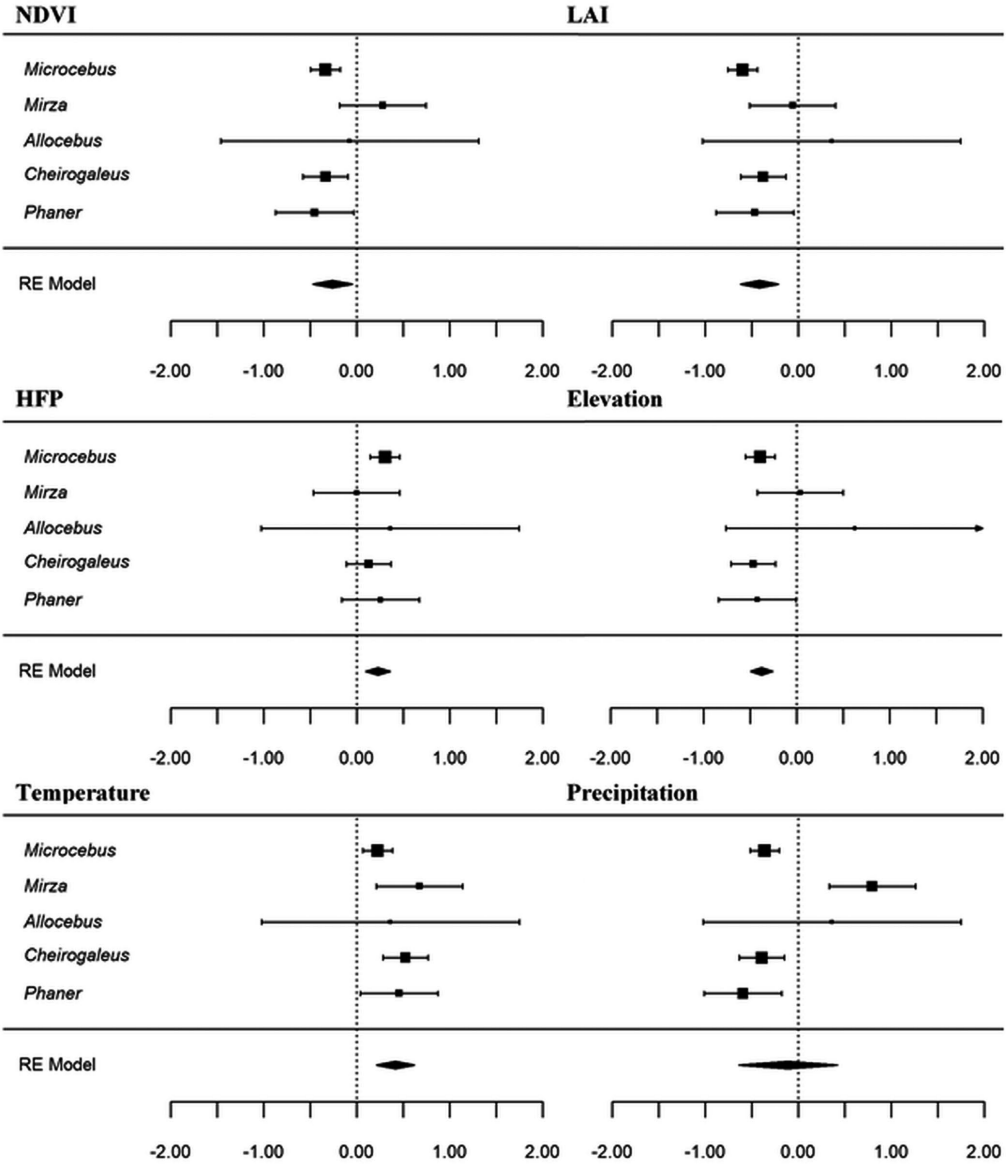
Forest plots of effect sizes with 95% confidence intervals (horizontal bars) from genus‐level meta‐analyses of the relationship between the population density of the five cheirogaleid genera and NDVI, LAI, HFP, elevation, temperature, and precipitation. The size of the effect (square) for each genus is proportional to that of the sample size

The meta‐analyses showed that the Cheirogaleidae family as a whole had a significantly negative correlation with NDVI, LAI, and elevation, but a strong positive correlation with HFP and temperature (Figure 1, Appendix [Link], [Link], [Link], [Link], [Link]). The direction of the correlations varied considerably among each specific genus (Figure 1). *Microcebus* density correlated positively with HFP and temperature, and negatively with NDVI, LAI, elevation, and precipitation. *Mirza* density correlated positively with both climatic variables but had no significant correlation with NDVI, LAI, HFP, and elevation. *Cheirogaleus* density correlated positively with temperature, negatively with NDVI, LAI, elevation, and precipitation and not significantly at all with HFP. *Phaner* correlations mirrored those of *Cheirogaleus*, while *Allocebus* density did not correlate significantly with any variable. The *Q* tests of heterogeneity suggest that there is significant variability between the five cheirogaleid genera for precipitation (*Q* = 27.734, *df* = 4, *p* < 0.001), but for none of the other variables. Pairwise comparisons of the *Z*‐coefficient 95% confidence intervals of each genus revealed minimal significant differences between genera across all six variables (Appendix S5). However, some significant differences did exist between genera for NDVI (*Microcebus* with *Mirza*) and precipitation (*Mirza* with *Microcebus*, *Phaner,* and *Cheirogaleus*) (Appendix S5).

### 3.2 Primary Density Drivers

**FIGURE 2:**
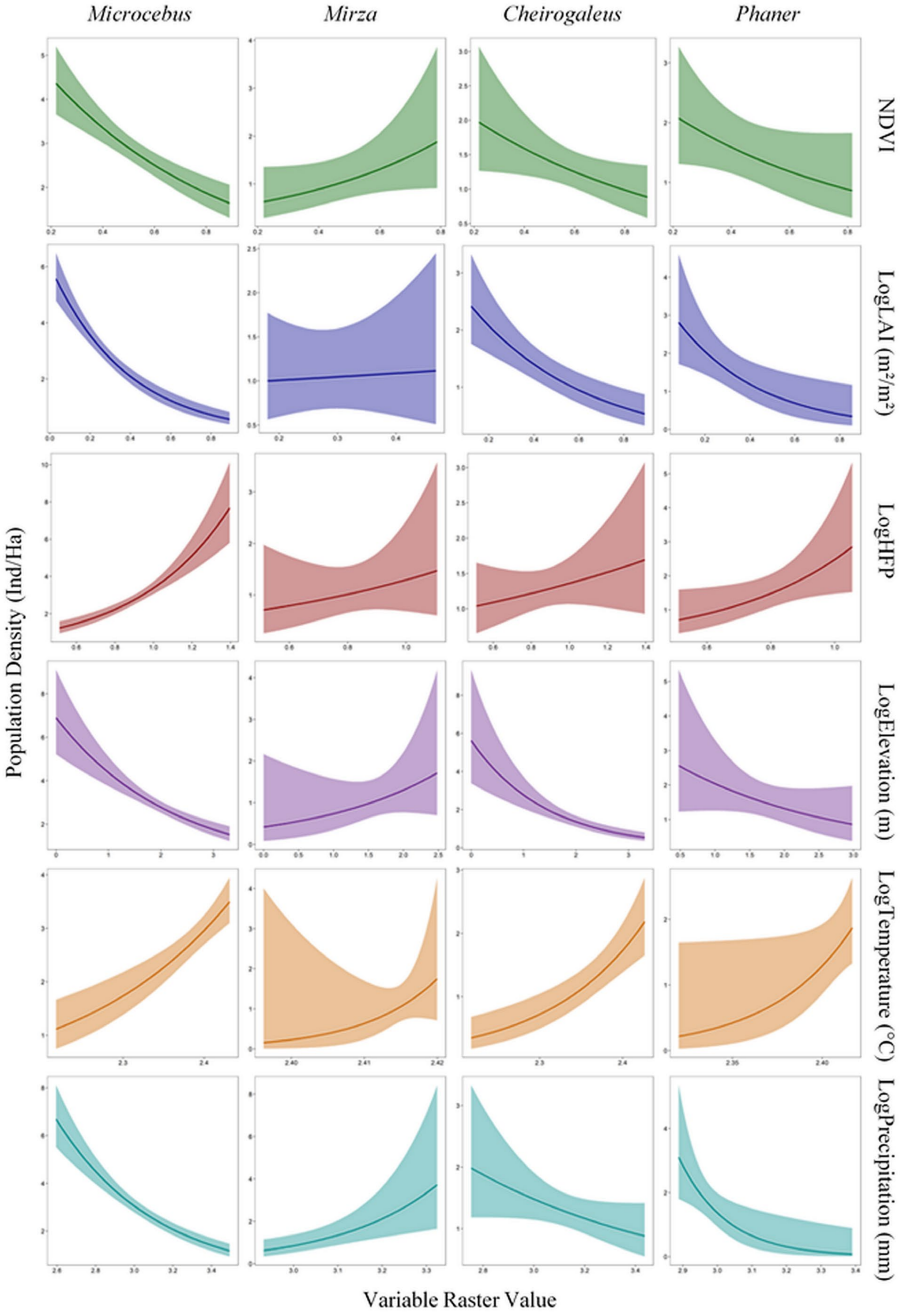
GLMM model curves (with 95% confidence intervals) of the relationship *of Microcebus* (column 1)*, Mirza* (column 2)*, Cheirogaleus* (column 3), and *Phaner* (column 4) population density with NDVI (green), LAI (dark blue), HFP (red), elevation (violet), temperature (orange), and precipitation (light blue) in Madagascar. Variable units are stated with the exception of the NDVI and HFP variables whose units are arbitrary. Curves were plotted using the R package “ggplot2” (Wickham, 2016)

**FIGURE 3:**
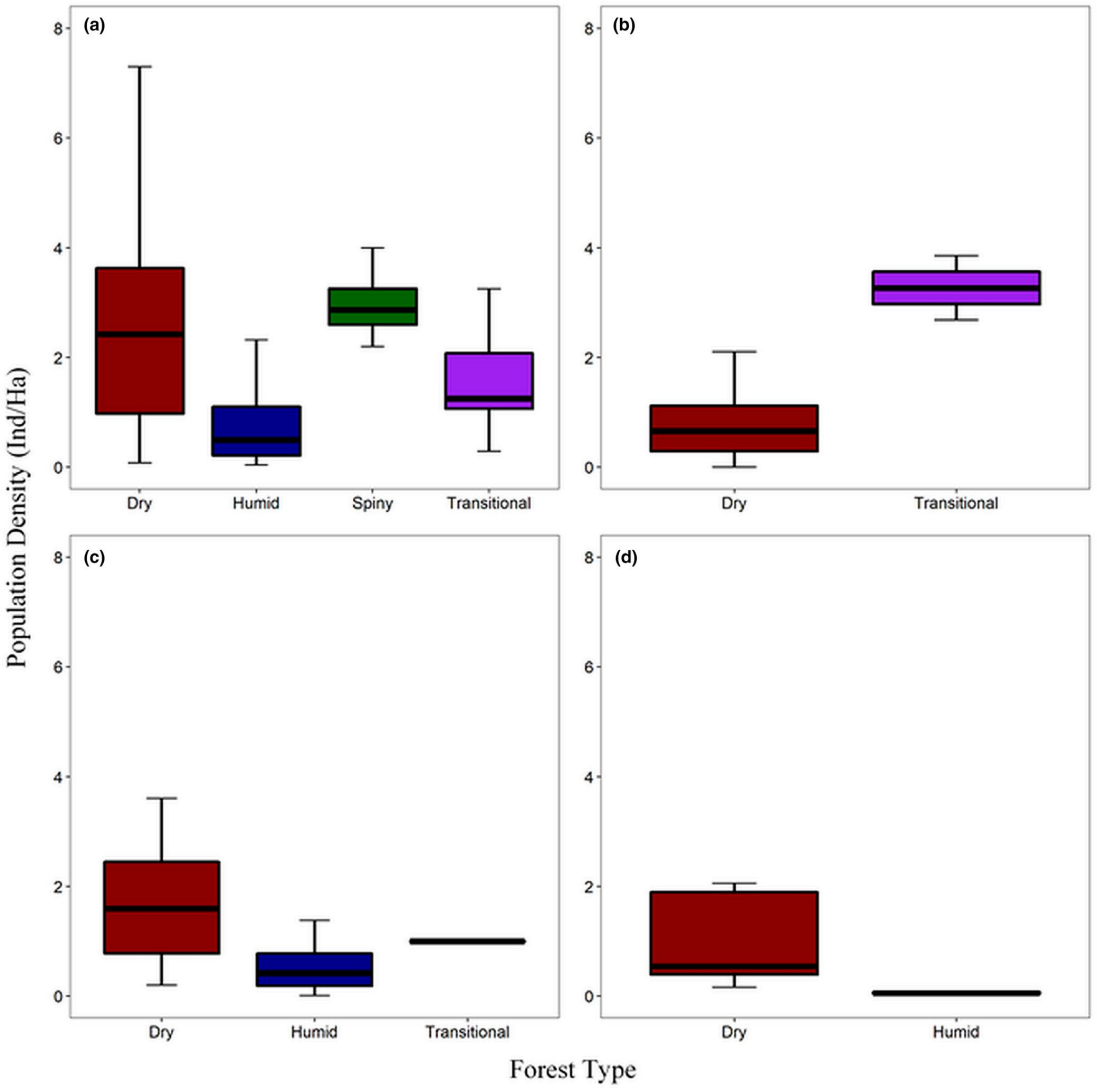
A comparison of how population density of the Cheirogaleidae varies between the four major forest types of Madagascar (Chauvet, 1972). A: *Microcebus*, B: *Mirza*, C: *Cheirogaleus*, D: *Phaner*. Only forest types for which density data exists for each genus are included in each plot. Figure was created using the R package “ggplot2” (Wickham, 2016)

The results of the GLMMs revealed that NDVI, LAI, and HFP are the strongest predictors of *Microcebus* population density, of which NDVI and LAI has a negative influence while HFP has a positive influence (Figure 2, Appendix S3). The climatic variables appear to be the strongest predictors of *Mirza* density, with both variables having a positive influence on density (Figure 2, Appendix S3). Temperature also appears to be the primary driver of *Phaner* density, having a positive influence on this genus, but there appears to be no primary environmental driver of *Cheirogaleus* population density (Figure 2, Appendix S2). Although the model curves show that the population density of all four genera had both positive and negative relationships with all environmental variables (Figure 2, as also shown in Figure 1), the results of the GLMMs show that many of these relationships were not significant when accounting for the effects of all other independent variables and the random effects (Appendix S3). This was also the case for forest type for all four genera (Figure 3, Appendix S3), although densities of *Mirza* are generally higher in transitional forest than in dry forest (Figure 3b). The variance of season, sampling method, and density calculation method was minimal, and lemur detectability (forest type) had no significant effect on the models (Appendix 3).

### 3.3 Influence of Protected Areas

**FIGURE 4:**
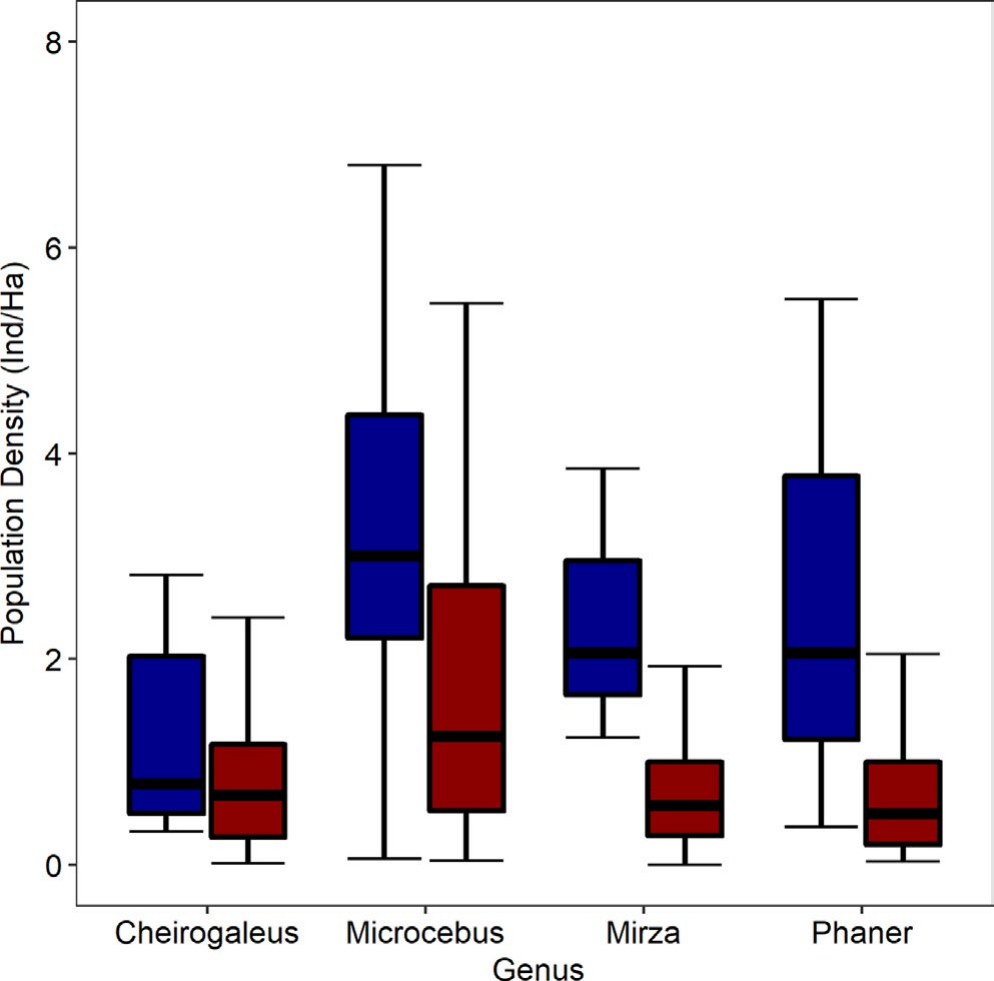
A comparison of how population density of *Microcebus*, *Mirza*, *Cheirogaleus*, and *Phaner* varies between protected areas (red) and unprotected areas (blue). Figure was created using the R package “ggplot2” (Wickham, 2016)

The Levene tests revealed that the datasets of each genus were all of homogenous variance and were thus suitable for GLM analysis (Appendix S4). Overall, *Microcebus* (*F*_1,148_ = 10.614, *p* =.001) and *Mirza* (*F*_1,15_ = 9.113, *p* =.009) population densities were significantly higher in unprotected areas than in protected areas (Figure 4). However, there was no significant difference between protected and unprotected areas for the population densities of *Cheirogaleus* (*F*_1,62_ = 0.703, *p* =.405) and *Phaner* (*F*_1,19_ = 1.108, *p* =.306) (Figure 4).

## 4 DISCUSSION

The results of this study revealed that the relationships between individual environmental variables and population density, and the primary drivers of these densities, were variable among the five genera as per my initial hypothesis (Figures 1, 2, 3).

### 4.1 Genus‐Specific Density Relationships with Environmental Variables

#### 4.1.1 Microcebus

*Microcebus* density correlated negatively with both NDVI and LAI (Figure 1), suggesting that population densities are generally higher in areas of lower vegetation availability and less dense tree cover. The meta‐analysis also revealed a surprisingly positive relationship with HFP (Figure 1). Although sensitivity to anthropogenic disturbance has been observed in *Microcebus* in rare instances (Schäffler & Kappeler, 2014), many species thrive in disturbed areas (Knoop et al., 2018) and anthropogenic disturbance (HFP) and forest quality (NDVI and LAI) are often strongly correlated (Liira et al., 2007), explaining these observations for *Microcebus*. *Microcebus* density correlated positively with temperature yet negatively with both elevation and precipitation (Figure 1). Changes in elevation often correlate with climate, and negative relationships with elevation have already been observed for several lemur species, including many of the Cheirogaleidae (Campera et al., 2020; Goodman & Ganzhorn, 2004). While the results for *Microcebus* reflect these findings, the correlations with elevation and climate could be due to higher densities of *Microcebus* in western dry forests than in eastern humid forests, as observed by Setash et al. (2017, but see Figure 3a); humid forests are at higher elevations and have lower mean temperatures yet higher annual precipitation than western dry forest. *Microcebus* do undergo daily torpor during their diurnal resting periods (Schmid, 2000), and some species can even undergo seasonal torpor to survive periods of environmental hardship (Atsalis, 1999). Heterothermy is therefore a notable component of their ecology, and this could provide an alternate explanation for the positive correlation that *Microcebus* have with temperature.

Unlike the other cheirogaleid lemurs, vegetation availability and quality (Figure 2, Appendix S3) and anthropogenic disturbance appear to be the primary driver of *Microcebus* density, as is the case for many other groups of animals globally (Bender et al., 1998; Schwitzer et al., 2011). However, for *Microcebus* the trend appears to be the opposite of most other animal groups, and they actually appear to have a positive relationship with anthropogenic disturbance and a negative relationship with forest quality, which contrasts with my original prediction. Further, *Microcebus* density does not appear to vary significantly between any of the forest types of Madagascar (in contrast to Setash et al., 2017). These findings highlight the remarkable adaptability and plasticity that *Microcebus* has to different habitat types and the habitat degradation and fragmentation ongoing throughout Madagascar, and it underpins their resilience reported in numerous other studies (Kappeler & Rasoloarison, 2003; Lehman et al., 2016; Schüßler et al., 2018). However, as all lemurs depend on forest for their survival, these relationships are only true if forest habitat is available. Also, while mouse lemurs have highly similar general ecologies and natural histories, each species within the *Microcebus* genus occupies its own ecological niche (Kamilar et al., 2016) and some species are more specialized than others (e.g., Kamilar et al., 2016; Radespiel et al., 2012).

#### 4.1.2 Mirza

*Mirza* density had no significant relationship with either NDVI, LAI, and HFP (Figure 1). As cheirogaleid lemur abundance and density are often unaffected by habitat quality and vegetation availability (Ganzhorn, 1995; Lehman et al., 2006a; Sawyer et al., 2017), this result is therefore not surprising, although it opposes my original prediction. The lack of correlation between density and HFP is also expected, as cheirogaleid lemurs have frequently been observed to persist and even thrive in anthropogenic habitats (Ganzhorn, 1987; Hending et al., 2018; Webber et al., 2020). *Mirza* density had no correlation with elevation but was positively correlated with both temperature and precipitation (Figure 1), which appear to be the primary drivers of *Mirza* density (Figure 2). This finding may also explain the higher densities of *Mirza* in transitional forests, which have higher annual rainfalls yet retain the high temperatures that are characteristic of dry forests (Chauvet, 1972).

The GLMM indicated that forest quality and anthropogenic disturbance do not have a significant influence on the density of *Mirza* (Figure 2, Appendix S3). As this genus contains only two species, this lack of significance is very unlikely to be the result of interspecies differences in habitat preference within the genus. Further, both species have been observed in highly degraded habitats in previous studies (LaFleur, 2020; Webber et al., 2020). Many Cheirogaleidae are adaptable and resilient to changes in habitat and are able to survive in a range of habitat types and in highly degraded areas (Forbanka, 2018a; Lahann, 2008; Mittermeier et al., 2010); the results of this study clearly demonstrate this for *Mirza,* which suggests that the two *Mirza* species are ecological generalists. While my results suggest that temperature and precipitation are the primary drivers of *Mirza* density, there are no aspects of this genus’ ecology and natural history that explain these findings, especially considering that *Mirza* do not undergo daily or seasonal torpor (Rode‐Margono et al., 2016).

#### 4.1.3 Cheirogaleus

Similar to *Microcebus*, *Cheirogaleus* density correlates negatively with both NDVI and LAI and therefore contradicts my original hypothesis. These findings reflect previous studies in which the abundance and density of *Cheirogaleus* in degraded areas is consistent to (or higher than) that of primary forest (e.g., Murphy et al., 2016; Hending, Andrianiaina et al., 2017, but see Andrianasolo et al., 2006). Also similar to *Microcebus*, *Cheirogaleus* density correlated positively with temperature yet negatively with both elevation and precipitation (Figure 1). The strong positive relationship that *Cheirogaleus* (and to some degree *Microcebus*) density has with temperature is particularly interesting as this genus undergoes periods of hibernation, and temperature is therefore a fundamental determinant of their activity patterns and ecology (Dausmann & Blanco, 2016). However, it is unexpected that *Cheirogaleus* density correlated negatively with precipitation, as *Cheirogaleus* often enter hibernation to survive periods of lower fruit availability induced by seasonal decreases in precipitation (Dausmann & Blanco, 2016); densities would be expected to be higher in areas with more rainfall. Although not yet investigated, *Cheirogaleus* density may mirror the east–west disparities that can be observed in *Microcebus* (Setash et al., 2017), with higher densities in the western dry forests than in the east (suggested in Figure 3c). If this is the case, then this would explain the relationships between *Cheirogaleus* density and elevation, temperature, and precipitation observed in this study.

The *Cheirogaleus* genus as a whole did not have any identifiable density drivers (Figure 2, Appendix S3). This is likely due to interspecific variation in ecological niches and habitat preferences within the genus; species are generally restricted to either dry, humid, or transitional forest types (density did not differ significantly between forest types, Figure 3c) and the genus contains some more‐generalist species (e.g., *C. medius*) and some taxa that are more specialized due to their geographic restriction within an altitudinal range (Blanco et al., 2009). Further, some *Cheirogaleus* live in sympatry (e.g., Blanco et al., 2009; Lahann, 2008), and the ecological and climatic niche separation that exists among these species on a local scale to permit their coexistence would make it very difficult to determine the primary density drivers for the genus as a whole (Kamilar & Muldoon, 2010). *Cheirogaleus* survive the cooler, dry season in prolonged hibernation, and heterothermy is thus a crucial component of their ecology (Dausmann & Blanco, 2016). It is highly likely that temperature and climate, or other weather‐related factors such as frost and water availability (Axel & Maurer, 2011), are major drivers of *Cheirogaleus* distribution and density, but as hibernation patterns are interspecific (Dausmann & Blanco, 2016), the directionality of species‐specific density‐climate correlations may oppose each other, making this effect detectable at the species level only.

#### 4.1.4 Phaner

*Phaner* density correlated negatively with both NDVI and LAI. While lower NDVI and LAI localities may not provide much shelter (Ganzhorn & Schmid, 1998), these sites sometimes have a larger availability of gum trees which are a primary food source of *Phaner* (Ganzhorn, 1995; Génin, 2008). Sites with low NDVI and LAI may therefore be able to support higher densities of these lemurs. As with *Microcebus* and *Cheirogaleus*, *Phaner* density correlated positively with temperature yet negatively with both elevation and precipitation (Figure 1). While a negative relationship between density and precipitation could be explained by a generalist ecology and therefore an ability to survive in harsh climates and a range of habitats (Kamilar & Muldoon, 2010), this observation is most probably due to the correlation that habitat quality and vegetation cover would have with precipitation. Further, *Phaner* are known to have a highly specialized gummivorous diet despite there being limited data on their ecology (Charles‐Dominique & Petter, 1980), and thus, they should not be considered as ecological generalists. *Phaner* has a negative relationship with forest quality (represented here by NDVI and LAI, Figure 1), and this would also explain the negative relationship with precipitation.

Temperature appears to be the primary driver of *Phaner* density, and while my comparisons of densities between forest types did not reflect this (Figure 3d), *Phaner* densities have been observed to be higher in dryer forests with higher temperatures (Forbanka, 2018b). Although *Phaner* are known to occupy transitional forest (Groves & Tattersall, 1991), only density data from dry forests (*P. pallescens*) and humid forests (*P. furcifer*) were available in the literature. Population densities and species richness of lemurs and other mammal species are often higher in Madagascar's hotter, dry forest habitat than in the cooler, humid forests (Muldoon & Goodman, 2015; Setash et al., 2017), and *Phaner* also appears to follow this pattern. However, as with *Mirza*, there are no aspects of *Phaner* ecology to explain their positive density relationship with temperature (Charles‐Dominique & Petter, 1980).

#### 4.1.5 Allocebus

*Allocebus* density did not correlate significantly with any of the environmental variables in the meta‐analyses (Figure 1). This was due to the small sample size of density values (*N* = 5) that exist in the literature, resulting in large 95% confidence intervals and limited statistical power for the meta‐analyses to detect any correlations of significance. The small sample size of *Allocebus* also prevented any analysis of the primary density drivers, and density comparisons between Madagascar's forest types were not conducted as *Allocebus* has only been confirmed in humid forest. Much more data are needed before any conclusions can be made about the effect of environment on *A*. *trichotis* density, the only species of this genus, although such data may be difficult to collect due to the elusiveness of this species (Meier & Albignac, 1991).

### 4.2 Influence of Protected Areas on Cheirogaleidae Population Density

The positive and negative effects of anthropogenic disturbance and forest quality on the densities of the Cheirogaleidae genera is further reflected in my comparison of densities between protected and unprotected areas (Figure 4). *Microcebus* and *Mirza* population densities appear significantly higher in unprotected areas in comparison with protected areas (Figure 4). However, the results for *Cheirogaleus* and *Phaner* were not statistically significant, even though their mean population densities were also higher in unprotected areas (Figure 4). As all lemurs depend on forest for their survival (Schwitzer et al., 2013), the GLM results for *Microcebus* and *Mirza* are highly unexpected, as most deforestation and land conversion in Madagascar has historically occurred and is currently occurring (mostly) in unprotected areas (Goodman et al., 2018; Harper et al., 2007). However, Madagascar's unprotected areas do still contain many forest fragments and gallery forests within the anthropogenic grassland matrix (Eklund et al., 2016; Goodman et al., 2018), and our current knowledge of *Microcebus* and *Mirza* indicate that many species are easily capable of maintaining healthy, viable populations within these unprotected areas (Lehman et al., 2016). Further, the GLMM and meta‐analyses results show that *Microcebus* may have preference for the degraded, anthropogenic habitats typical of Madagascar's unprotected areas, and these unprotected areas may in fact be a more suitable habitat type for them. As with the GLMM analysis, comparisons could not be made for *Allocebus* as no records of *A. trichotis* in unprotected areas exist. This is because it either cannot survive in these areas, or it has simply not yet been surveyed for and observed there; this species may rely on the higher quality habitat of protected areas for survival in contrast to *Microcebus* and *Mirza* (as also suggested in the meta‐analyses: Figure 1). In comparison with the Cheirogaleidae, many diurnal/cathemeral lemurs depend on protected areas for their survival and thus maintain high population densities within the large, continuous forests of protected areas (Ganzhorn et al., 2000; Schwitzer et al., 2013). While there may be higher competition for resources amongst lemurs in protected areas, the Cheirogaleidae occupy different ecological and temporal niches to potential competitors (Donati et al., 2013; Ganzhorn, 1989). In addition, many cheirogaleid species live sympatrically alongside many other lemur species, often at high densities (e.g., Lehman et al., 2006a; Ralison, 2008), suggesting that the higher densities of *Microcebus* and *Mirza* in unprotected areas cannot be attributed to competition pressures within protected areas.

### 4.3 Limitations and Future Directions

Although the population abundance and density of cheirogaleid lemurs has been investigated in many studies, my literature review revealed that the way in which the results are reported varies considerably. While many studies report on actual population density values, almost half of the data points that I found in the literature (49.2%) reported encounter rates, and were thus not comparable with true density values. The sample sizes for some genera were therefore low, which limited the statistical power of my meta‐analyses. Also, the method of data collection and density calculation varied between studies included in my dataset, although I was able to control for this, and for season and differences in lemur detectability between forest types, in the analyses that I used. This highlights the need for a standard protocol for the reporting of population density and abundance values so that Metadata can be more easily and consistently compared. Furthermore, some individual species have no data concerning their distribution and population density at all, either because they have only been described very recently or because they have remained unstudied (Lehman et al., 2016). While this had no bearing on this genus‐level study, many of these species are already listed as threatened on the IUCN Red List (IUCN, 2020), and researchers should prioritize obtaining this information that is vital for the conservation and management of their populations (Schwitzer et al., 2013).

This study has revealed some information on how three biotic and three abiotic variables determine the population density of the Cheirogaleidae. However, several other factors that were not possible to include in this study have also been documented to influence lemur density, distribution, and abundance. These include forest edge proximity (Lehman et al., 2006a), vegetation structure (Rendigs et al., 2003), plant nutritional quality (Simmen et al., 2012), and predation pressure (Karpanty, 2006). While data for these variables are difficult to collect, standardize, and include in studies such as this, efforts should be made to expand our knowledge of how these additional factors may also influence the cheirogaleid meta‐population density.

### 4.4 Conservation Implications and Conclusion

The overall findings of this study suggest that the Cheirogaleidae, particularly *Microcebus*, are highly adaptable and resilient to the ongoing habitat degradation and anthropogenic disturbance associated with Madagascar's high rates of deforestation and forest fragmentation (Harper et al., 2007). My results also suggest that some Cheirogaleidae, such as *Microcebus* and *Mirza*, may not be fully dependent on Madagascar's protected area system, which is encouraging for the conservation of these threatened lemurs. However, all lemurs, including the Cheirogaleidae, require forest habitat to survive, and some diurnal and cathemeral lemurs heavily depend on protected areas and continuous forests for survival (Ganzhorn et al., 2000; Schwitzer et al., 2013, but see LaFleur & Gould, 2009; Donati et al., 2011; Gould & Gabriel, 2015; Eppley et al., 2017). The most effective way to maintain Madagascar's forest habitat and mitigate deforestation is through the protective legislation that the protected area system offers. Madagascar's protected area network is therefore crucial for the conservation of all lemurs and many other threatened and endemic species, despite the encouraging results of my study. Further, new‐growth secondary forests resulting from the extensive reforestation and habitat restoration regimes taking place throughout the island are likely to play a vital role in species conservation, if deforestation and habitat fragmentation continues in Madagascar at its current rate. Finally, obtaining the population data that is missing for as‐yet unstudied species should be a high conservation priority so that the populations of all species can be effectively monitored and species‐specific conservation action plans can be implemented to ensure their survival (Schwitzer et al., 2013).

To conclude, this study of the Cheirogaleidae has highlighted that different environmental factors can influence the population densities of very‐closely related animals in very different ways. Additionally, some environmental factors can more strongly determine population density than others, and density–environment correlates are not always as expected and do not always conform to regular hypotheses (as highlighted here by higher *Microcebus* and *Mirza* densities in unprotected areas). Knowledge of these mechanisms is thus of vital importance to fully understand the biogeography and ecology of animals, to determine their ecological niches, and to implement successful conservation of their populations.

## CONFLICT OF INTEREST

None declared.

## AUTHOR CONTRIBUTION

**Daniel Hending:** Conceptualization (lead); Data curation (lead); Formal analysis (lead); Investigation (lead); Methodology (lead); Project administration (lead); Resources (lead); Software (lead); Validation (lead); Visualization (lead); Writing‐original draft (lead); Writing‐review & editing (lead).

## Supporting information

Appendix S1

Appendix S2

Appendix S3

Appendix S4

Appendix S5

## Data Availability

The data presented in this paper are available on Dryad: https://doi.org/10.5061/dryad.4mw6m908x
