# Supplementary material for: Environmental drivers of Cheirogaleidae population density: Remarkable resilience of Madagascar’s smallest lemurs to habitat degradation
Source: Ecol Evol. 2021 May 2;11(11):5874–91. doi: 10.1002/ece3.7449 (PMC8207435; doi:10.1002/ece3.7449)
Supplement: Supplementary file 1 — Appendix S1 [file ECE3-11-5874-s004.docx]

Literature

1. Andrianarivo, AJ. (1981) *Etude comparée de l'organisation sociale chez Microcebus coquereli.* Unpublished Dissertation, University of Madagascar, Antananarivo*.*
2. Ausilio, E. and Raveloanrinoro, G. (1998) Les lémuriens de la région de Bemaraha: Forêts de Tsimembo, de l’Antsingy et de la région de Tsiandro. *Lemur News*. 3, pp. 4-7.
3. Blanco, MB., Rasoazanabary, E. and Godfrey, LR. (2015) Unpredictable environments, opportunistic responses: Reproduction and population turnover in two wild mouse lemur species (*Microcebus rufus* and *M. griseorufus*) from eastern and western Madagascar. *American Journal of Primatology*. 77(9), pp. 936-947.
4. Blow, R., Fisher, J., Foglietti, CB., Wintersgill, D., Cornfoot, R., Gré du Haut, Razafindrakoto, RGH., Tsiafa, HA., Andriatahinjanahary, RSG., Ferguson, B. and Ratsirarson, J. (2014) Abundance of five nocturnal species and the influence of habitat characteristics in the littoral forest of Tampolo, northeast Madagascar. Lemur News. 18, pp. 52-57.
5. Bohr, YEMB., Giertz, P., Ratovonamana, YR. and Ganzhorn, JU. (2011) Gray-brown mouse lemurs (*Microcebus griseorufus*) as an example of distributional constraints through increasing desertification. *International Journal of Primatology*. 32(4), pp. 901-913.
6. Bousquet, B. and Rabetaliana, H. (1992) Site du patrimoine mondial des Tsingy de Bemaraha et autres sites d’intérêt biologique et écologique du fivondronana d’Antsalova. Rapport UNESCO, Paris.
7. Charles-Dominique, P. and Petter, JJ. (1980) Ecology and social life of Phaner furcifer. In *Nocturnal Malagasy Primates: Ecology, Physiology and Behavior* (Charles-Dominique, P., Cooper, HM., Hladik, A., Hladik, CM., Pages, E., Pariente, GF., Petter-Rousseaux, A., Petter, JJ. and Schilling, A. Eds.). pp.75-96. Academic Press, New York.
8. Donati, G. (2002) *L’attività e le sue correlate ecologiche nel lemure bruno dal collare, Eulemur fulvus collaris (Lemuridae), nella foresta litorale di Ste Luce (FortDauphin, Madagascar)*. Unpublished PhD thesis, Università di Pisa.
9. Evans, MI., Thompson, PM. and Wilson, A. (1995) A survey of the lemurs of Ambatovaky Special Reserve, Madagascar. *Primate Conservation*. 14, pp. 13-31.
10. Fietz, J. (1999) Demography and floating males in a population of *Cheirogaleus medius*. In *New Directions in Lemur Studies* (Rakotosamimanana, B., Rasamimanana, H., Ganzhorn, JU. and Goodman, SM. Eds.). pp. 159-172. Springer, Boston, MA.
11. Fleagle, JG., Janson, CH. and Reed, KE. (1999) Spatial and temporal scales in primate community structure. In *Primate Communities* (Fleagle, JG., Janson, CH. and Reed, KE. Eds). pp. 284–288. Cambridge University Press, Cambridge, UK.
12. Ganzhorn, JU. (1988) Food partitioning among Malagasy primates. *Oecologia*. 75(3), pp. 436-450.
13. Ganzhorn, JU. and Kappeler, PM. (1996) Lemurs of the Kirindy forest. *Primate Report.* 46(1), pp. 257-274.
14. Gardner, CJ., Fanning, E., Thomas, H. and Kidney, D. (2009) The lemur diversity of the Fiherenana-Manombo Complex, southwest Madagascar. *Madagascar Conservation and Development*. 4(1), pp. 38-43.
15. Génin, F. (2008) Life in unpredictable environments: first investigation of the natural history of *Microcebus griseorufus*. *International Journal of Primatology*. 29(2), pp. 303-321.
16. Hawkins, AFA., Chapman, P., Ganzhorn, JU., Bloxam, QMC., Barlow, SC. and Tonge, SJ. (1990) Vertebrate conservation in Ankarana special reserve, northern Madagascar. *Biological Conservation*. 54(2), pp. 83-110.
17. Herrera, JP., Wright, PC., Lauterbur, E., Ratovonjanahary, L. and Taylor, LL. (2011) The effects of habitat disturbance on lemurs at Ranomafana National Park, Madagascar. *International Journal of Primatology*. 32(5), pp. 1091-1108.
18. Hladik, CM., Charles-Dominique, P. and Petter, JJ. (1980) Feeding strategies of five nocturnal prosimians in the dry forest of the west coast of Madagascar. *Nocturnal Malagasy Primates: Ecology, Physiology, and Behaviour* (Charles-Dominique, P., Cooper, HM., Hladik, A., Hladik, CM., Pages, E., Pariente, GF., Petter-Rousseaux, A., Petter, JJ. and Schilling, A. Eds.). pp.41-73. Academic Pres, New York.
19. Jolly, A. (1987) Priorites dans l'etude des populations de Lemuriens. In: *Priorities en Matiere de Conservation des Espices a Madagascar* (Mittermeier, RA., Rakotovao, LH., Randrianasolo, V., Sterling, EJ. and Devitre, D. Eds.). Occasional Papers of the IUCN Species Survival Commission, Number 2.
20. Jolly, A. (1988). Lemur survival. In *Primates: The Road to Self-Sustaining Populations* (Benirschke, K. Ed.). pp. 71-98. Springer-Verlag, New York.
21. Lehman, SM., Ratsimbazafy, J., Rajaonson, A. and Day, S. (2006) Ecological correlates to lemur community structure in southeast Madagascar. *International Journal of Primatology*. 27(4), pp. 1023-1040.
22. Lehman, SM., Rajaonson, A. and Day, S. (2006) Lemur responses to edge effects in the Vohibola III classified forest, Madagascar. *American Journal of Primatology*. 68(3), pp. 293-299.
23. Lehman, SM. and Wright, PC. (2000) Preliminary study of the conservation status of lemur communities in the Betsakafandrika region of eastern Madagascar. *Lemur News*. 5, pp. 23-25.
24. Lehman, SM., Rajaonson, A. and Day, S. (2005) Composition of the lemur community in the Vohibola III Classified Forest, SE Madagascar.  *Lemur News.*10, pp.16-19.
25. Meyler, SV., Salmona, J., Ibouroi, MT., Besolo, A., Rasolondraibe, E., Radespiel, U., Rabarivola, C. and Chikhi, L. (2012) Density estimates of two Endangered nocturnal lemur species from Northern Madagascar: New results and a comparison of commonly used methods. *American Journal of Primatology*. 74(5), pp. 414-422.
26. Miller, A., Mills, H., Ralantoharijaona, T., Volasoa, NA., Misandeau, C., Chikhi, L., Bencini, R. and Salmona, J. (2018) Forest type influences population densities of nocturnal lemurs in Manompana, northeastern Madagascar. *International Journal of Primatology*. 39(4), pp. 646-669.
27. Müller, P., Velo, A., Raheliarisoa, EO., Zaramody, A. and Curtis, DJ. (2000) Surveys of sympatric lemurs at Anjamena, north‐west Madagascar. *African Journal of Ecology*. 38(3), pp. 248-257.
28. Norscia, I., Rahanitriniaina, OG., Alison, J. and Giuseppe, D. (2006) Preliminary survey of lemur density in the semimontane rainforest of Anka, Fort-Dauphin region. *Lemur News*, 11, pp. 14-17.
29. Petter, JJ. (1978) Ecological and physiological adaptations of five sympatric nocturnal lemurs to seasonal variations in food production. *Recent Advances in Primatology* (Chivers, D. Ed.). pp. 211-223. Academic Press.
30. Petter, JJ. and Petter-Rousseaux, A. (1964) Première tentative d’estimation des densités de peuplement des Lémuriens malgaches. *La Terre et la Vie*. 4, pp. 427-435.
31. Petter, JJ., Schilling, A. and Pariente, G. (1971) Observations Eco éthologiques sur deux lémuriens malgaches nocturnes: *Phaner furcifer* et *Microcebus coquereli*. *La Terre et la Vie*. 25, pp. 287-327.
32. Pollock, JI. (1979) Spatial distribution and ranging behaviour in lemurs. In *The Study of Prosimian Behaviour* (Doyle, GA. and Martin, RD. Eds.), pp. 359-409. Academic Pres, New York.
33. Rabeson, P., Randrianarisata, D., Rasabo, P., Andrianoely, D., Razafindrakoto, G., Razafindraibe, D., Rasabo, L. and Wright, PC. (2006) Surveys for lemurs and biodiversity in the Beakora forest southeast of Kalambatritra Reserve, Madagascar. *Lemur News*. 11, pp. 5-9.
34. Radespiel, U. (2000) Sociality in the gray mouse lemur (*Microcebus murinus*) in northwestern Madagascar. *American Journal of Primatology*. 51, pp. 21–40.
35. Radespiel, U., Ehresmann, P. and Zimmermann, E. (2001) Contest versus scramble competition for mates: the composition and spatial structure of a population of gray mouse lemurs (*Microcebus murinus*) in north-west Madagascar. *Primates*. 42(3), pp. 207-220.
36. Rakotondratsimba, G., Ralisoamalala, R. and Ratsimbazafy, JH. (2013) Les lémuriens du site Ramsar de Torotorofotsy. *Madagascar Conservation and Development*. 8(1), pp. 29-38.
37. Rakotondravony, R. and Radespiel, U. (2009) Varying patterns of coexistence of two mouse lemur species (*Microcebus ravelobensis* and *M. murinus*) in a heterogeneous landscape. *American Journal of Primatology*. 71(11), pp. 928-938.
38. Ralison, JM. (2006) A lemur survey of the Réserve Spéciale de Marotandrano, Madagascar. *Lemur News*. 11, pp. 35-37.
39. Ralison, J. (2006b) Rapid assessment of lemurs in southern and southwestern forests of Madagascar. *Lemur News*. 11, pp. 35-38.
40. Ralison, JM. (2007) Lemur survey of the Andranoma-nitsy Forest, region of Besalampy, Province of Mahajanga. *Lemur News*. 12, pp. 36-39.
41. Ralison, JM. (2008) Les lémuriens des forets sèches malgaches. *Les forêts sèches de Madagascar. Malagasy Nature*. 1, pp. 135-156.
42. Randrianambinina, B., Rasoloharijaona, S., Rakotondravony, R., Zimmermann, E. and Radespiel, U. (2010) Abundance and conservation status of two newly described lemur species in northwestern Madagascar (*Microcebus danfossi, Lepilemur grewcockorum*). *Madagascar Conservation and Development.* 5, pp. 95–102.
43. Rasoarimanana, J. (2005) Suivie de lémuriens diurnes dans le Parc National de Andohahela. *Lemur News*. 10, pp. 27-29.
44. Rasolofoson, D., Rakotondratsimba, G., Rakotonirainy, O., Rasolofoharivelo, T., Rakotozafy, L., Ratsimbazafy, J., Ratelolahy, F., Andriamaholy, V. and Sarovy, A. (2007) Le bloc forestier de Makira charnière de Lémuriens. *Lemur News*. 12, pp. 49-53.
45. Rasolofoson, D., Rakotondratsimba, G., Rakotonirainy, O., Rakotozafy, LM., Ratsimbazafy, JH., Rabetafika, L. and Randrianarison, RM. (2007b) Influences des pressions anthropiques sur les lémuriens d’Anantaka, dans la partie est du plateau de Makira, Maroantsetra, Madagascar. *Madagascar Conservation and Development*. 2(1), pp. 21-27.
46. Reed, KE. (1999) Population density of primates in communities: differences in community structure. In *Primate Communities* (Fleagle, JG., Janson, CH. and Reed, KE. Eds). pp. 116-140. Cambridge University Press, Cambridge.
47. Safford, RJ. and Duckworth, JW. (1990) A wildlife survey of Marojejy nature reserve. International Council for bird preservation.
48. Salmona, J., Rakotonanahary, A., Thani, IM., Zaranaina, R., Ralantoharijaona, T., Jan, F., Rasolondraibe, E., Barnavon, M., Beck, A., Wholhauser, S. and Ranirison, P. (2014b) Estimation des densités et tailles de population du Microcèbe Roux du Nord de (*Microcebus tavaratra*) dans la région Loky-Manambato (Daraina). *Lemur News*. 18, pp. 73-75.
49. Sawyer, RM., Fenosoa, ZSE., Andrianarimisa, A. and Donati, G. (2017) The effect of habitat disturbance on the abundance of nocturnal lemur species on the Masoala Peninsula, northeastern Madagascar. *Primates*. 58(1), pp. 187-197.
50. Schäffler, L. and Kappeler, PM. (2014a) Distribution and abundance of the world's smallest primate, *Microcebus berthae*, in central western Madagascar. *International Journal of Primatology*. 35(2), pp. 557-572.
51. Schäffler, L. and Kappeler, PM. (2014b) Distribution and abundance of three cheirogaleid species in Menabe Central, Western Madagascar. *Lemur News*. 18, pp. 38-43.
52. Schüßler, D., Radespiel, U., Ratsimbazafy, JH. and Mantilla-Contreras, J. (2018) Lemurs in a dying forest: Factors influencing lemur diversity and distribution in forest remnants of north-eastern Madagascar. *Biological Conservation*. 228, pp. 17-26.
53. Schwab, D. and Ganzhorn, JU. (2004) Distribution, population structure and habitat use of Microcebus berthae compared to those of other sympatric cheirogaleids. *International Journal of Primatology*. 25(2), pp. 307-330.
54. Steffens, TS. and Lehman, SM. (2016) Factors determining *Microcebus* abundance in a fragmented landscape in Ankarafantsika National Park, Madagascar. In *The Dwarf and Mouse Lemurs of Madagascar: Biology, Behavior and Conservation Biogeography of the Cheirogaleidae* (Lehman, S., Radespiel, U. and Zimmermann, E. Eds.) pp. 477-498. University Press, Cambridge.
55. Sterling, E. and McFadden, K. (2000) Rapid census of lemur populations in the Parc National de Marojejy, Madagascar. *Fieldiana Zoology*. 97, pp.265-274.
56. Sterling, EJ. and Rakotoarison, N. (1998) Rapid assessment of richness and density of primate species on the Masoala peninsula, eastern Madagascar. *Folia Primatologica*. 69(Suppl. 1), pp. 109-116.
57. Sterling, EJ. and Ramaroson, MG. (1996) Rapid assessment of the primate fauna of the eastern slopes of the Reserve Naturelle Integrale d'Andringitra, Madagascar. *Fieldiana Zoology*. 85, pp. 293-305.
58. Weidt, A., Hagenah, N., Randrianambinina, B., Radespiel, U. and Zimmermann, E. (2004) Social organization of the golden brown mouse lemur (*Microcebus ravelobensis*). *American Journal of Physical Anthropology*. 123(1), pp. 40-51.
59. Wright, PC., Erhart, EM., Tecot, S., Baden, AL., Arrigo-Nelson, SJ., Herrera, J., Morelli, TL., Blanco, MB., Deppe, A., Atsalis, S. and Johnson, S. (2012) Long-term lemur research at Centre ValBio, Ranomafana National Park, Madagascar. In *Long-term Field Studies of Primates* (Kappeler, PM. and Watt, DP. Eds.). pp. 67-100. Springer, Berlin, Heidelberg.

**Literature without Density Values (only encounter rates)**

1. Forbanka, DN. (2018) Population surveys of fork-marked dwarf lemurs and needle-clawed galagos. *Primates*. 59(4), pp. 355-360.
2. Hawkins, AFA. (1999) The primates of Isalo National Park, Madagascar. *Lemur News*. 4, pp. 10-14.
3. Hending, D., Andrianiaina, A., Rakotomalala, Z. and Cotton, S. (2017) Range extension and behavioural observations of the recently described Sheth's dwarf lemur (*Cheirogaleus shethi*). *Folia Primatologica*. 88(5), pp. 401-408.
4. Hending, D., Andrianiaina, A., Rakotomalala, Z. and Cotton, S. (2018) The use of vanilla plantations by lemurs: encouraging findings for both lemur conservation and sustainable agroforestry in the Sava region, northeast Madagascar. *International Journal of Primatology*. 39(1), pp. 141-153.
5. Irwin, MT., Smith, TM. and Wright, PC. (2000) Census of three eastern rainforest sites north of Ranomafana National Park: preliminary results and implications for lemur conservation. *Lemur News*. 5, pp. 20-22.
6. Irwin, MT., Samonds, KE. and Raharison, JL. (2001) A biological inventory of the lemur community of Réserve Spéciale de Kalambatritra, south-central Madagascar. *Lemur News*. 6, pp. 24-28.
7. Lehman, SM., Rajaonson, A. and Day, S. (2006) Edge effects on the density of Cheirogaleus major. *International Journal of Primatology*. 27(6), pp. 1569-1588.
8. Malone, M., Ramanamanjato, JB., Randriatafika, F. and Donati, G. (2013) Habitat structure and grey mouse lemur (*Microcebus murinus*) abundance in the transitional littoral forest of Petriky, South-East Madagascar. *Lemur News.* 17*,* pp. 22-26*.*
9. Markolf, M., Kappeler, PM. and Rasoloarison, R. (2008) Distribution and conservation status of *Mirza zaza*. *Lemur News*. 13, pp. 37-40.
10. Nash, L. (2000) Encounter rate estimates on *Lepilemur leucopus* and *Microcebus murinus* at Beza Mahafaly Special Reserve, southwestern Madagascar. *Lemur News*. 5, pp. 38-40.
11. Olivieri, G., Craul, M. and Radespiel, U. (2005) Inventaire des lémuriens dans 15 fragments de forêt de la province de Mahajanga. *Lemur News*. 10, pp. 11-16.
12. Rakotondravony, R. and Rabenandrasana, M. (2012) Inventaire des lémuriens dans la zone de Pointe à Laree, Soanierana-Ivongo, Région Analanjirofo, Madagascar: Implication pour la conservation. *Lemur News*. 16, pp. 43-48.
13. Ralantoharijaona, T., Besolo, A., Rabarivola, CJ., Schwitzer, C. and Samona, J. (2014) Densité de la population de Lepilemur mittermeieri dans la région d’Ampasindava (Nord-Ouest de Madagascar). *Lemur News*. 18, pp. 7-10.
14. Randrianambinina, B. and Rasoloharijaona, S. (2006) Inventaires des lémuriens nocturnes dans la forêt pluviale de Maromizaha (Est de Madagascar). *Lemur News*. 11, pp. 9-12.
15. Rasoloharijaona, S., Randrianambinina, B., Rakotosamimanana, B. and Zimmermann, E. 2005. Inventaires des lémuriens dans la forêt d'Andranovelona/Madirovalo (nord ouest de Madagascar), les" savoka" dé Manehoko, la Réserve de Lokobe, la Réserve Spéciale de l'Ankarana, et le Réserve Spéciale d'Analamerana, au nord de Madagascar. *Lemur News*. 10, pp. 8-11.
16. Webber, AD., Solofondranohatra, JS., Razafindramoana, S., Fernández, D., Parker, CA., Steer, M., Abrahams, M. and Allainguillaume, J. (2020) Lemurs in Cacao: Presence and Abundance within the Shade Plantations of Northern Madagascar. *Folia Primatologica*. 91(2), pp. 96-107.
